# Supplementary material for: The effect of systemic administration of salicylate on the auditory cortex of guinea pigs
Source: PLoS One. 2021 Nov 11;16(11):e0259055. doi: 10.1371/journal.pone.0259055 (PMC8584678; doi:10.1371/journal.pone.0259055)
Supplement: Attachment — Submitted filename: Response to Reviewers.docx [file pone.0259055.s004.docx]

September 20, 2021

Susan E Shore, Ph.D.
Academic Editor
*PLOS ONE*

Dear Editor:

We wish to re-submit the attached manuscript titled “**The effect of systemic administration of salicylate on the auditory cortex of guinea pigs**.” The manuscript ID is PONE-D-21-16756.

We thank you and the reviewers for your thoughtful suggestions and insights, which have substantially enriched the manuscript. We hope that the revised manuscript is now suitable for publication in your journal.

The manuscript has been rechecked and appropriate changes have been made in accordance with the reviewers’ suggestions. The responses to their comments have been prepared and attached herewith. The revisions are indicated in red font in the revised manuscript.

Thank you for your consideration. We look forward to hearing from you.

Sincerely,

Kentaro Ochi, MD

Department of Otolaryngology, St. Marianna University School of Medicine

2-16-1, Sugao, Miyamae-ku, Kawasaki City, 216-8511

Fax: +81-44-977-8111

Tel. no.: +81-44-977-8111

E-mail: [k2ochi@w6.dion.ne.jp](mailto:k2ochi@w6.dion.ne.jp)

Reviewer 1

Thank you for reviewing our manuscript.

We have carefully considered the comments provided by you and the other reviewer, and the manuscript was revised accordingly.

Major

1. We have added the analysis of frequency tuning by measuring the Q_10dB_ value.

2. We have not recorded the peripheral auditory system in this experiment. In the previous study, we administered salicylate at a dose of 100 mg/kg. In the present study, we applied 200 mg/kg. We believe that the dose is enough for inducing changes.

3. We have changed the figure accordingly.

4. We have added a new figure about the CF distribution of two areas.

5. We have separately added a figure about threshold change with respect to time.

6. We have added a figure about spontaneous activity change with respect to time.

7. Following your comment, we have deleted the sentence.

Minor:

1. We have revised the title and abstract to indicate that salicylate was systemically administered.

2–7. Following your suggestion, we have revised the sentences.

8. We have changed the sentences and added a figure accordingly.

9. We have changed the sentences as suggested.

Reviewer 2

Thank you for reviewing our manuscript.

We have carefully considered the comments provided by you and the other reviewer, and the manuscript was revised accordingly.

1. We have changed the explanation of the result and legend.
2. We have added a figure and descriptions about single unit separation. We continuously recorded the same single unit throughout the experimental session. We confirmed waveforms to check for single unit separation by choosing clusters.
3. We have applied the Friedman test for the analysis.
4. Out point was not used for analysis. In addition, it was used not as a numeric value but as an inverted rank.

5. It depends on the animal stock. The animals were 3 weeks old upon arrival. We usually experiment during 2–10 weeks after their arrival. We intended to minimize influences, such as age, animal’s condition, condition of the experimental room, anesthesia, etc.; therefore, we continuously recorded the same single unit throughout the experiment. We mainly applied the paired test for the analysis instead of using a non-paired test.

6. We have deleted the sentences as suggested.
